# PRRs and NB-LRRs: From Signal Perception to Activation of Plant Innate Immunity

**DOI:** 10.3390/ijms20081882

**Published:** 2019-04-16

**Authors:** Ali Noman, Muhammad Aqeel, Yonggen Lou

**Affiliations:** 1 Institute of Insect Sciences, College of Agriculture and Biotechnology, Zhejiang University, Hangzhou 310027, China; 2 Department of Botany, Government College University, Faisalabad 38000, Pakistan; 3 State Key Laboratory of Grassland Agro-ecosystems, School of Life Science, Lanzhou University, Lanzhou 730000, China; aqeelbutt99@gmail.com

**Keywords:** defense, plants, PAMPs, pathogenesis, transcriptional activity

## Abstract

To ward off pathogens and pests, plants use a sophisticated immune system. They use pattern-recognition receptors (PRRs), as well as nucleotide-binding and leucine-rich repeat (NB-LRR) domains, for detecting nonindigenous molecular signatures from pathogens. Plant PRRs induce local and systemic immunity. Plasma-membrane-localized PRRs are the main components of multiprotein complexes having additional transmembrane and cytosolic kinases. Topical research involving proteins and their interactive partners, along with transcriptional and posttranscriptional regulation, has extended our understanding of R-gene-mediated plant immunity. The unique LRR domain conformation helps in the best utilization of a surface area and essentially mediates protein–protein interactions. Genome-wide analyses of inter- and intraspecies PRRs and NB-LRRs offer innovative information about their working and evolution. We reviewed plant immune responses with relevance to PRRs and NB-LRRs. This article focuses on the significant functional diversity, pathogen-recognition mechanisms, and subcellular compartmentalization of plant PRRs and NB-LRRs. We highlight the potential biotechnological application of PRRs and NB-LRRs to enhance broad-spectrum disease resistance in crops.

## 1. Introduction

Like animals, plant immune responses depend on cellular events, but plants possess a pathogen-recognition system to balance the absence of an adaptive immune system [1,2,3]. The plant immune system properly identifies and tackles pathogens and related pathogenesis events [3,4,5]. Studies focusing on plants under pathogen attack have improved our knowledge of plant–pathogen interactions and offer novel crop-protection strategies. Pathogens prefer host cells as source of the nutrients required for their growth. After pathogen recognition, plant cells broadly reprogram their metabolic activities and switch on their defense mode.

Plant immunity comprises two tiers [6,7]. Primarily, pathogen-associated molecular patterns (PAMPs), microbial-associated molecular patterns (MAMPs), or damage-associated molecular patterns (DAMPs) are recognized by host surface receptor proteins called pattern-recognition receptors (PRRs). PRRs can be categorized as plasma-membrane-localized receptor kinases (RKs) or receptor-like proteins (RLPs). RKs possess an ectodomain for binding with ligands, a single transmembrane domain, and an intracellular kinase domain. RLPs are deficient in apparent intracellular signaling domains. More than 600 receptor-kinase genes and 57 receptor-like proteins have been reported in the *Arabidopsis* genome. Among these, many are involved in biotic stress responses [8,9]. The nucleotide-binding leucine-rich repeats (NB-LRR) gene family is one of the largest gene families in the Plantae kingdom. NB-LRR genes are found as isolated genes as well as in clusters of different sizes. NB-LRRs are devoid of definite kinase domains and found attached to additional domains [10,11,12]. On the basis of the amino terminus, NB-LRRs are divided into two major groups, i.e., toll interleukin 1 receptor NB-LRR (TIR-NB-LRR) and coiled-coil-NB-LRR (CC-NB-LRR).

PAMP/MAMP detection by PRRs initiates physiological modulations in the cell. Then, a cascade of response begins that, through the identification of PAMPs or MAMPs, results in plant immunity known as PAMP-triggered immunity (PTI) [13]. Very soon after PAMPs recognition, signaling events activate pathogen-related responses such as an increase in cytosolic Ca^2+^, reactive-oxygen-species (ROS) production, and kinase activation, i.e., calcium-dependent (CDK) and mitogen-activated (MAPK), protein phosphorylation, and variations in gene regulation for the production of antimicrobial compounds [14,15,16]. If the first line of defense is surpassed by pathogens, then plant resistance initiates a second line of defense, Effector-Triggered Immunity (ETI). PTI is suppressed by effector proteins injected by pathogens into the plant cell [17]. Cytosolic recognition of these pathogenic effectors by NB-LRR proteins activates ETI. *R* genes encode NB-LRRs proteins and leucine-rich repeats (LRR) are responsible for their specific binding interactions [7,10]. ETI displays coevolutionary dynamics in plant–pathogen interaction [18]. Contrary to PAMPs, effectors are typically variable and dispensable. In comparison with PTI, ETI is qualitatively strong, rapid, and often result in a hypersensitive response (HR) [19]. A comparison of both immunity tiers shows that ETI performs well against adapted pathogens in nonhost resistance. However, it has clearly been demonstrated that ETI, PTI, and basal defense share signaling mechanisms [20,21,22]. In a single plant species, approximately 20% of all genes respond to pathogen recognition with coordinated changes in their expression [23,24].

In this article, we summarize the roles of PRRs and NB-LRRs in connection with PTI and ETI for plant defense. Recent progress in the field of plant innate immunity was highlighted, and we tried to bridge the gaps in understanding plant defense responses. We unraveled the interesting functions of PRRs and NB-LRRs, along with more information about effector molecules and their recognition, subcellular localization, as well as the suppression of PTI responses. As a final point, we briefly discuss how this knowledge loops into crop protection that helps in exploring plant defense actions.

## 2. PRRs, the Welcome Receptionists, and PTI

PRRs can be categorized as plasma-membrane-hosted RKs or RLPs. RKs consist of an ectodomain for ligand binding, a single transmembrane domain, and an intracellular kinase domain. [25]. Receptor-like proteins are devoid of any obvious indigenous signaling domain [26]. Previous studies showed that PAMPs are recognized with the help of PRRs (Table 1) [27]. This PAMP recognition was well-described in the case of the *Arabidopsis* receptor kinase FLS2 (Flagellin Sensing 2) that directly binds with bacterial flagellin and forms a signaling complex [28,29,30]. After elicitation, FLS2 interacts with Brassinosteroid Insensitive Associated Kinase 1 (BAK1) and forms a protein complex. This interaction causes protein phosphorylation. BAK1 may perceive some other elicitors by heterodimerization with PRRs in the LRR-receptor kinase family [31]. We demonstrate that pathogen mediated upregulation of Botrytis-Induced Kinase 1 (BIK1) potentially regulates the FLS2–BAK1 complex. Prior reports confirm BIK1–FLS2–BAK1 interaction before elicitation, and BIK1 probably dissociates from the FLS2–BAK1 complex after elicitation. In vitro analysis revealed that BIK1 phosphorylates both FLS2 and BAK1. In the same way, BAK1 is needed for PTI upon identification of elf18, a peptide derived from bacterial elongation factor EF-Tu, by the elongation factor receptor (EFR). Induction of PTI is supported by the fact that both FLS2 and EFR are members of the RLK superfamily, possessing the extracellular LRR domain intracellular kinase domain [32]. More evidence comes from PEPRs-1 and PEPRs-2 (Pep receptors that belong to RLK) that recognize Peps in *Arabidopsis*. Both of these resemble FLS2 and EFR in structure and also need BAK1 to dispatch Pep signals. All endogenous elicitors like Peps are termed together as DAMPs [32,33]. It is likely that PTI-based responses in the case of viral pathogens, where no conserved PAMP has been reported so far, can be triggered via DAMP activation. Despite the positive contribution of BAK1 in plant immunity, some contrasting results with particular reference to BAK1 are also on record. For example, in comparison with wild-type plants, BAK1 mutant *Arabidopsis* plants showed resistance against *Pseudomonas syringae* by the overproduction of salicylic acid (SA) [34]. On the other hand, the same mutant plants exhibited high susceptibility to *Botrytis cinerea*. A closer look at the literature highlights deficiencies in FLS2-mediated immunity, and, from these findings, it is difficult to ascertain the clear role of BAK1 in plant–pathogen interactions. Therefore, extensive studies are required to determine the exact role of PRRs and different molecular regulators during plant defense.

The *Xa21* gene in rice codes for an RLK that confers resistance against *Xanthomonas oryzae* pv *oryzae*. *Xa21*-mediated immunity is activated by an avirulence protein corresponding to the XA21 protein (avrXa21), now renamed as Ax21 (activator of Xa21-mediated immunity) [35]. Numerous bacterial genes needed for Ax21 activity were recognized for encoding constituents of a bacterial type I secretion system or for their involvement in sulfation. axYS22 (sulfated 17-amino acid synthetic peptide), derived from Ax21, was adequate for biological activity and binding with Xa21. On the other hand, nonsulfated peptides remained incapable of performing biological activity. This axYS22 is found in all *Xanthomonas* species [36,37,38]. Similarly, Pep-13 is a narrowly conserved PAMP in *Phytophthora* sp. (Table 1) [35,39]. The currently accepted knowledge is that PAMPs are considered to be widely conserved across genera, whereas effectors are specific to single or a few related species [7,39,40]. For instance, chitin is a unanimously agreed PAMP from fungi. Similarly, cell-wall β-glucan and elicitins from oomycetes, as well as bacterial elongation factors, flagellin, peptidoglycans, lipopolysacharides are recognized PAMPs that induce PTI. Likewise, lipopeptides from Mycoplasma, phosphatidyl-myo-inositol mannosides from Mycobacteria, and oligomannosides in viruses also act as PAMPs [41]. There are several exceptions to this model reviewed by Thomma et al. [39]. They indicated that some PAMPs are narrowly conserved, while some effectors show wide distribution. So, we are in a position to say that different PAMPs display a varied and wide distribution spectrum. Interestingly, only some effector proteins on the basis of their widespread distribution e.g., LysM effectors, can be designated as PAMPs. In light of the zig-zag model, we can anticipate some fungal effectors are well able to meddle with immunity triggered by chitin. Ecp6, an effector from *Cladosporium fulvum*, also may suppress host immunity triggered by chitin [42]. LysM effectors from fungal pathogens may contribute in virulence [39]. Their functional conservation in the Fungi kingdom is suggestive of archetypal PAMPs. Contrarily, Avr4 effector homologs from *Cercospora* do not bind with chitin. This highlights differential binding capacity between different effector groups and clearly reflects effector race specificity.

It is pertinent to mention that not all PAMPs are widely recognized. For example, some PAMPs, such as EF-Tu and cold-shock protein, are only recognized by limited plant hosts belonging to plant families Brassicaceae and Solanaceae, respectively [43,44]. This fact was further substantiated in *Nicotiana benthamiana* that cannot perceive EF-Tu. Transiently expressed receptor kinase EFR helped *N. benthamiana* plants in acquiring EF-Tu perception by binding to EF-Tu binding sites [6,45]. Notably, several microbial patterns play the role of PAMPs in plants, but their corresponding binding sites have not been explored. We imply that appropriate ligand recognition by immune receptors triggers plant resistance, and the intensity of the defense response can be dependent upon the requisite level of actual immunity.

## 3. PRRs May Undergo Homodimerization, Heterodimerization, or Heteromultimerization

Ligand recognition by PRRs results in PTI [25]. Receptor dimerization/polymerization is essential for the initiation of signaling and triggering plant immune responses. AtCERK1, a homolog of OsCEBiP, possesses three lysine motif (LysM) extracellular domains to bind chitin oligomers. Likewise, LysM RLK1 is also necessary for chitin-triggered immunity [76]. CERK1 (Chitin Elicitor Receptor Kinase 1) has a role in identifying bacterial peptidoglycans (PGNs) for mediating immunity in *Arabidopsis thaliana* [75]. From these facts, we point out that the binding of CERK with chitin or PGNs is, in fact, the ability to perceive completely different pathogens, i.e., fungi or bacteria. In addition to a role in plant immunity, proteins containing LysM distinguish chitin-related molecules as well as Nod factors for the initiation of root nodulation [77]. We argue that, before the initiation of immune signaling, the homodimerization of CERK1 is induced by chitin residues acting as bivalent ligands and forms an active receptor complex. This receptor complex triggers immune signaling induced by long-chain chitin molecules. From these facts, it is clear that the binding between small chitin oligomers (4–5 GlcNac residues) and CERK1 may take place, but such complexes are unable to induce CERK1 homodimerization and ultimately fail to trigger defense responses [76,78].

Binding between chitin and AtCERK1 results in the phosphorylation of the intracellular kinase domain and triggers disease resistance [74,79,80]. Therefore, chitin-mediated oligomerization is indispensable for CERK1 activation and signaling initiation, and offers a baseline to understand PAMP-induced PRR activation. Just like *Arabidopsis* CERK1, CEBiP (Chitin Oligosaccharide Elicitor Binding Protein) in rice homodimerizes before immune signaling. Hayafune and colleagues [81] proposed that two CEBiP molecules simultaneously bind to a chitin oligosaccharide from the opposite side. This binding causes the dimerization of CEBiP in rice. The C-terminal of CEBiP does not possess signaling motifs for other RLPs, which proposes its function in signal initiation with the help of additional proteins [72]. Indeed, OsCEBiP forms a hetero-oligomeric receptor complex with OsCERK1 in the presence of chitin [82]. Unlike AtCERK1, OsCERK1 possesses an extracellular LysM domain needed for chitin-mediated signaling and not for binding chitin [82]. Therefore, we inferred that chitin perception in rice is considerably different from *Arabidopdsis*. Rice defense essentially requires a hetero-oligomeric receptor complex [83]. In contrast to OsCEBiP, AtCEBiP is not mandatory for traditional chitin immune responses [84]. Faulkner et al. [85] reported that AtLYM2 (the closest ortholog of AtCEBiP) is involved in the CERK1-independent and chitin-induced closure of plasmodesmata against fungal attacks. Consequently, it can be concluded that certain chitin-triggered cellular responses in *A. thaliana* require hetero-oligomerization between a chitin-binding AtLYM and a yet-unknown RLK possibly linked with CERK1.

For some other RLKs, ligand-induced heterodimerization has been elaborated [86,87]. Research has provided evidence for bacterial flagellin (flg22) perception in *Arabidopsis* mediated by FLS2 [28,88]. Although FLS2 is conserved among different plant species, perception of flg22 among plants shows differences. For example, AtFLS2 expression conferred an extra flg22-perception system in tomatoes that resembled in properties with the perception of flg22 in *Arabidopsis* [88]. In short, FLS2 is the PRR that determines the specificity of flg22 perception. FLS2 and BAK1 are closely located on plasma membrane, and BAK1 acts as a coreceptor for flg22. Here, two important things are noteworthy. First, after binding flg22 with FLS2, its ectodomain interacts with the ectodomain of BAK1 and, second, the C-terminal of flg22, bound to FLS2, stabilizes FLS2–BAK1 dimerization. This C-terminal region of flg22 actually functions as the molecular glue for joining both ectodomains. So, we can say that FLS2–BAK1 heterodimerization is mediated by receptor as well as ligand. A similar activation mechanism in *Arabidopsis* was observed for RLK–LRR–BRI1 (Brassinosteroid Insensitive 1) and BAK1/SERK1 (somatic embryogenesis receptor kinases) [89]. This proposes the involvement of many LRR-containing RLKs (and RLPs) in the same heterodimeric complexes with BAK1 or related SERK proteins [90].

## 4. Pathogen Effectors and PTI Suppression

Over the course of evolution, pathogens developed the ability to surpass PTI and cause acute damage [26,91]. A critical open question is how PTI is surpassed. Normally, phytopathogens tackle PTI by means of effectors [92], which can be quite variable. Such specificity/variability can be evidenced even among strains of a species. For instance, two *Pseudomonas* strains, i.e., S1E40 and S3E12, significantly differ from each other in the organization of the type-III secretion system (T3SS) and effector proteins that ultimately affect their capability to induce HR in plants. Collectively, 565 and 567 effector proteins were identified in the S1E40 and S3E12 strains, respectively. Genomic analysis revealed one nonflagellar and two flagellar-based T3SS clusters in the genomes of both strains [93]. A large number of existing studies in the broader literature revealed that 20–30 effectors can be injected by means of T3SS, e.g., *P. syringae* [94,95]. Just like bacterial T3SS, nematodes inject effectors via stylet into the apoplast, or through feeding tubes directly into the cytosol [96]. Fungal and oomycete effectors seemingly move by the eukaryotic (type II) secretory pathway. This pathway is actually the exocytosis of Golgi-derived secretory vesicles. Most fungi and oomycete effectors possess an N-terminal type II secretion signal that is essentially needed to cross the first two boundaries [97,98].

In light of the reported findings, it is confirmed that T3SS is necessary for pathogen infection because bacterial mutants devoid of T3SS show nonpathogenic characteristics due to failure in effector-mediated interference with PTI [99]. We can then affirm that PTI suppression is directly linked with bacterial pathogenicity [100]. Of note is that not all effectors target PTI, e.g., *Xanthomonas* transcription activator-like (TAL) effectors induce host genes that take part in disease-symptom development [92]. Additionally, TALs are required for virulence and do not act redundantly. A DNA binding domain is essentially needed for the interaction between TAL and the target gene promoter [101]. Bacterial effectors are detected intracellularly by ETI receptors due to their specific molecular/enzymatic activities, e.g., AvrRpm1 and AvrB, are recognized by RPM1. Effector recognition by NB-LRRs can be direct or indirect. During direct effector recognition, an NB-LRR protein directly binds with the effector and activates ETI. During indirect binding, NB-LRRs interact with effectors and monitor it. Effectors like *AvrPtoB* adapt a dual tactic for kinase suppression. They are actually a part of bacterial strategy for the nonspecific targeting of host kinases [102,103,104]. *AvrPtoB* can target five host kinases of the Pto/interleukin receptor-associated kinase (IRAK) class, while *AvrPto* may act as kinase inhibitor. As this group is very large in plants, it is anticipated that *AvrPto* may target many more targets than these [102,105]. It is on record that an effector *HopF2* belonging to *P. syringae* may target RIN4. Bacteria without *HopF2* show enhanced growth in lines lacking RIN4 [106]. This proposes RIN4 as a target for virulence. However, this finding also has an indirect reason. The stomatal opening is an important event in leaf-based bacterial pathogenesis. Being a negative regulator of PTI as well as ETI, RIN4 also interacts with plasma membrane H^+^-ATPases, i.e., AHA1 and AHA, for increasing the stomatal opening [92,107]. Two different effectors, AvrRpm1 and AvrB, target *Arabidopsis* RIN4 and result in ETI that effectively curbs pathogen growth. Similarly, AvrRpt2 also targets *Arabidopsis* RIN4 and controls RPS2-mediated immunity. The effectors involved in this process are consistent with the fact that RIN4 is an important virulence target. The question is whether RIN4 targeting by some other proteins can also reduce bacterial virulence and increment plant defense. This issue needs to be extensively investigated.

The effector strategy for prokaryotic pathogens is strongly supported by much evidence. However, the question is whether eukaryotic pathogens adopt an effector strategy. Effectors in eukaryotic microbes such as fungi or oomycete are secreted by the endomembrane system and are consequently delivered into host cells [108]. An internal motif, i.e., Arg-X-Leu-Arg (RXLR, X is an amino acid), is required for the delivery of oomycete effectors into plant cells. These effectors are the result of extremely strong selection processes. The *Avr1d* gene in *Phytophthora sojae* encodes the RXLR effector protein. *P. sojae Avr* genes expressing effectors caused cell death in *Glycine max* plants that possess resembling *Rps* genes. Two Avr1d alleles activate ETI in *G. max* harboring the *Rps1d* gene. *P. sojae* strains lacking the *Avr1d* gene can overcome *Rps1d* [109]. RXLR effectors are well able to enter host cells, but it is not clear how this entry takes place. Presumably, binding to phospholipids may be the central point in targeting a host cell. It was proposed that RXLR motif functions in phosphatidylinositol 3-phosphate (PI3P) binding [110], but succeeding findings shows that the majority of lipid-binding affinity exists downstream in the carboxy (C)-terminal effector protein domain rather than the RXLR sequence [111,112]. With the help of several plant proteins containing the RXLR motif, many of which play role in membrane trafficking [113], it was proposed that oomycete effectors may gain entry into a host cell by using the plant endocytic pathway [108]. Certainly, in plant–rust interfaces, the tubular extensions of the extrahaustorial membrane are connected with budding vesicles that penetrate into the cytoplasm and come in close contact with thwe host endoplasmic reticulum and dictyosomes [114]. In a different situation, the parasite-derived protein channel is used for effector uptake [115]. Substantial developments in understanding eukaryotic and prokaryotic effectors propose the necessity to explore new and assorted roles of eukaryotic effectors to favor their specific nutrient-procurement tactics.

Detailed genomic and transcriptomic studies involving different pathogens and parasites, such as root knot nematodes, recognize many interesting aspects and elucidate the pathogenic strategies of these versatile organisms. Moreover, viral pathogens possess explicit suppressors of the sRNA pathway to avoid genome degradation and/or halt viral gene expression [116]. Apart from existing reports, knowledge about eukaryotic effectors and their targets is insufficient and not at an advanced stage. We argue that the previous literature suffers from certain weaknesses. Earlier studies can only be considered as an initial step toward a more profound understanding of eukaryotic effectors. Analyzing pathogenic effectors and their interactive partners is expected to recognize crucial elements in host defense mechanisms, immune pathways. and pathogenicity strategies.

## 5. NB-LRRs Recognize Pathogen Effectors by Direct or Indirect Interactions

**Figure 1:**
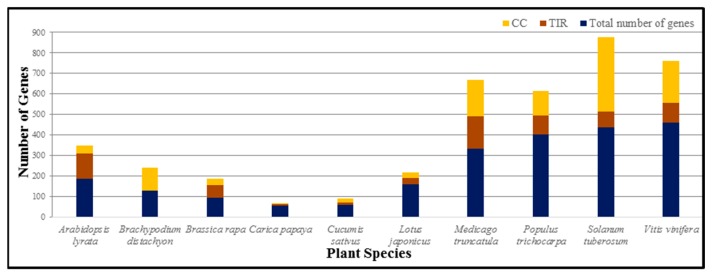
Presence of nucleotide-binding-site (NBS)-encoding R genes in different plants. Toll interleukin 1 receptor nucleotide-binding and leucine-rich repeat (TIR-NB-LRR) and coiled-coil-NB-LRR (CC-NB-LRR).

Effectors can be identified by NB-LRRs via direct protein–protein interactions or by detecting modifications in host proteins targeted by the effectors [117]. Effector proteins and their receptors exhibit diverse selection and have different recognition specificities [6,7,117]. We may attribute this to antagonistic coevolution between host interacting partners and pathogens. A large and growing body of literature has confirmed that nucleotide binding by the NB domain is required for the function of NB-LRR proteins in plants (Figure 1) [26,91]. Signal activation perhaps needs ATP and ADP in the binding site [118]. Effector interaction is mediated by LRR in recognition systems [92,119]. Oppositely, the NB-LRR can be autoinhibited during direct recognition [92]. Plant NB-LRR proteins confer resistance to varied pathogens, e.g., viruses, bacteria, and fungi (Table 2) [120,121].

NB-LRRs can identify many effectors rather than one. Without an effector trigger, NB-LRR proteins remained in confined conformation. NB-LRRs can be negatively regulated by the release of effector-associated accessory protein during indirect recognition before ETI activation [129]. The effector causes alterations in accessory proteins during indirect effector recognition. This change facilitates NB-LRRs in recognizing an accessory protein [130]. This tactic precisely points out the evolutionary benefit of rapidly evolving pathogens. It is certain that the host controls recognition by taking advantage of the pathogen’s virulence strategy. NB-LRRs belong to diverse families, inclusive of some members directly interacting with suitable effectors. Conversely, LRR domains of NB-LRRs involved in indirect recognition are frequently conserved [117]. Conserved and multidomain NB-LRR act as switch for the translation of different direct and indirect pathogen signals into an integrated immune response [131]. Ambiguities also persist about the part(s) of LRRs participating in indirect recognition of effectors. It is likely that direct and indirect recognition systems are involved in different NB-LRR activation methods. Different models have been proposed to explain effector recognition. It is pertinent to mention that proposed models are overviews based on limited specific evidence that are not yet completely understood. The great variety in effector–receptor studies proposes multiple variations on these ideas, and perhaps other novel recognition steps.

Although effector recognition by direct interaction is candid, it involves many R proteins for recognizing different effectors. This recognition can be overcome by pathogens by evolving new effector(s). Indirect effector recognition entails the detection of diverse effectors by a sole immune receptor without maintaining R protein collection, assisting in plant defense against pathogens [6]. This is helped by host proteins targeted directly by effectors or indirectly affected by their activities. Different reports have designated such immune-receptor-monitored host proteins as *guardee* or *decoy*. *Guardee* usually has an extra role in defense other than facilitating effector recognition [130]. RIN4, guarded by RPM1 and RPS2 (two NLRs), is the well-illustrated *guardee*. AvrB, AvrRpm1, and AvrRpt2, effectors of *Pseudomonas*, interact with RIN4. AvrB and AvrRPM1 induce changes in RIN4 phosphorylation and activate RPM1-mediated defense responses. RIN4 cleavage by AvrRpt2 triggers RPS2-mediated immune responses [132,133,134]. Here, we can observe that RIN4 is the focal point of three effectors that permit recognition of different effectors by a single NLR. The data recommend that some proteins like RPS4 and RRS1 make an authentic immune complex that dynamically deals with effectors and interact with immunity regulators, e.g., EDS1 (Enhanced Disease Susceptibility 1)/PAD4 [135]. These proteins work as part of a protein heterocomplex. Normally, one of the two member proteins in the immune complex plays the role of a sensor to detect effectors, while the other protein acts as a helper to trigger an immune response. However, we acknowledge that considerable discussions among researchers about the collective functioning of proteins remain to be explored. There are also multiple concerns to be addressed regarding recognition methods. Effector-based NB-LRR activation is a challenge. Therefore, it is imperative to understand similar/dissimilar activation mechanisms with respect to diverse recognition methods.

## 6. Can Pathogen Recognition by NB-LRRs Only Occur in the Nucleus?

Upon nuclear localization, NB-LRRs are activated and interact with nuclear factors to modify gene expression. For example, the N protein from *Nicotiana*, MlA10 from *H. vulgare*, and RPS4 in *A. thaliana* are found both in the cytoplasm and nucleus. Nuclear presence is essential for their functions [136,137]. It is important but very difficult to differentiate between interactions related to identification from those related to signaling. AtRRS1-R interacts with the PopP2 effector from *R. solanacearum* in the nucleus [138]. Common signaling components among different NB-LRR proteins are reportedly taking place inside the nucleus. It would be stimulating to determine the degree to which nuclear localization deals with signaling activity.

Different NB-LRRs have also been observed for their localization other than in the nucleus (Table 3). RPM1 is present in plasma membrane [139]. After acetylation, RIN4 (the guardee/host target protein) also localizes to plasma membrane. Because RPM1 is an exterior membrane-localized protein, it is essentially detained in place by binding to a different protein [100,140]. RIN4 may be that different protein used to detain RPM1. Hyperphosphorylation of RIN4 due to AvrRpm1 and AvrB may discharge RPM1 from the plasma membrane for the induction of immune signaling [140,141]. Interestingly, some NB-LRRs have been predicted for chloroplast localization that requires intensive investigation. “N”, a TIR-NB-LRR immune receptor, essentially needs a host factor NRlP1. This host factor is found exclusively in the chloroplast [142]. Over 50% of the TIR-NB-LRR family possesses chloroplast-targeting sequences. So, the question is whether pathogen recognition can take place in the chloroplast. Unequivocally, different secreted proteins from *Pseudomonas syringae* contain a chloroplast-targeting signal, e.g., HopI1 [143]. This effector modifies the ultrastructure of the chloroplast and hampers SA production that is a chloroplast-based defense signal [144]. Moreover, the photosystem II core complex is depleted during TMV [145]. Therefore, it is not unexpected if TIR-NB-LRRs recognize distresses inside the host chloroplast. The need is to inquire whether or not TIR-NB-LRRs can identify pathogens within chloroplasts. For triggering defense responses, such chloroplast-based recognition demands regressive signaling to the nucleus. NRIP1 in *Nicotiana* precisely interacts with the TIR domain of the N immune receptor. It would be motivating to check if NRIP1 can associate with chloroplastic TIR-NB-LRRs.

## 7. Signaling Components and Immune Responses

PTI and ETI are segregated immune responses but are part of defense actions during microbial infection [151,152,153]. Different cellular events, such as transcriptional reprogramming, calcium ion influx, ROS burst, MAPK activation (mitogen-activated protein kinases), and hypersensitive response are part of plant defense [153]. PTI and ETI responses display an analogy but differ in magnitude [154]. Understanding signaling events during plant immunity is essential. Although some diverse signaling components have been recognized, most such components of these pathways are obscure.

The SA, JA (jasmonic acid), and ET(ethylene) pathways are dynamic regulators of plant-defense gene expression [152,155]. Studies have proposed that these pathways act antagonistically to a little extent, i.e., SA provides resistance against biotrophic pathogens [152,155,156], while JA–ET helps in the defense against necrotrophic pathogens and insects. Many researchers have mentioned varied gene-expression outputs for these pathways [157]. It is pertinent to mention that numerous genes work as definite markers for activating the SA or JA–ET pathways. Some authors have confirmed that a PTI response is comparatively weak in comparison with ETI. According to Tsuda et al. [158], both the SA and JA–ET pathways appeared to work synergistically in PTI for amplifying defense responses, but the effector–target interaction suppresses PTI. Blocking only one component of these pathways is enough to markedly disturb the response. Nonetheless, the ETI response involves repeated activities of the SA as well as JA–ET pathways [158,159]. Consequently, in the case of no SA signaling, the JA–ET response supports plant resistance against pathogens, and a greater signal flux in ETI seemingly overwhelms this response against pathogenesis. In spite of modified gene expression due to PTI and ETI activation, key variations for the inhibition of pathogen growth are still not very clear in any disease system.

Some genes and signaling proteins suppress ETI. Enhance disease susceptibility 1 and Nonrace-specific disease resistance 1 (EDS1 and NDR1) are needed for TIR-NB-LRRs as well as CC-NB-LRRs signaling [160,161,162]; however, the connecting steps are not known. Missing signaling links propose essential elements in ETI signaling and the possibility of signaling pathways operating in parallel. Additional studies, i.e., biochemical approaches to better understanding and identifying the interactive partners of activated NB-LRR proteins are compulsory to unravel additional steps in these pathways.

Prominently, MAPKs are involved in PTI as well as ETI [163]. Ubiquitous MAPK transfers signals from extracellular receptors to the ultimate cellular response [2]. In *Arabidopsis*, a MAPK cascade acting downstream/upstream of flagellin perception activates the WRKY TF involved in plant defense [164]. Correspondingly, the constitutive function of MKK4 and MKK5 confers resistance against *P. syringae* in *A. thaliana*. MPK3, MPK6, and MPK4 are also activated in parallel cascades by PAMPs [2,165,166]. MPK3 and 6 are associated with the activation of numerous immune responses, and the inactivation of these MPKs compromises plant defense against pathogenesis. For example, upon flg22 perception, MPK6 phosphorylates 1-aminocyclopropane-1-carboxylic acid synthase (ACS6), leading to ethylene biosynthesis. Furthermore, ERF104 is a recognized MPK6 substrate. MPK6–ERF104 interaction seemingly allows the liberated ERF104 to activate ethylene signaling during PTI, and modulate the response to flg22 [167]. Likewise, the MEKK1–MKK1/2–MPK4 signaling framework performs critical functions in basal defense. Interruption in MAPK cascade leads to SUMM2 (Suppressor of MKK1 MKK2 2)-mediated constitutive defense responses. SUMM2, an NB-LRR protein, is an indirect protectant of MEKK1–MKK1/2–MPK4 activity by monitoring the status of CRCK3 phosphorylation (Calmodulin-Binding Receptor-like Kinase 3, a MPK4 substrate) [166]. CRCK3 is phosphorylated by MPK4 and binds with SUMM2, but it cannot trigger SUMM2-mediated immunity. By blocking of MPK4 activity, CRCK3 phosphorylation is decreased and results in SUMM2 conformational change for its activation. PAT1, another MPK4 substrate, also has a prominent role in SUMM2-mediated immunity [168]. The mechanism by which MEKK1–MKK1/2–MPK4 contributes to basal defense is still not very clear. CRCK3 mutant plants did not show more disease susceptibility [169] that proposes MPK4-based regulation of basal defense through proteins like MAP kinase substrate 1 (MKS1). This complicated protection mechanism for regulating the MAPK network through substrates displays versatility in comparison with protecting single proteins in the process. Several pathogen effectors target different modules of plant MAPK cascades. HopAI1-caused MPK4 inactivation elicits immune responses mediated by SUMM2 [166]. Independent of MAPKs, CDPKs are also essentially needed for FLS2-dependent immunity, while Ca^2+^ channel inhibitors abolish utmost immune responses caused by MAMPs or effectors. Together, these findings suggest intricate interplays between PAMP-triggered immunity and effector-triggered immunity via MAPK signaling.

## 8. Concluding Remarks

Global food security and price hikes for major agricultural crops have been in special focus for the last few decades. The soaring prices of food commodities and the demand–supply gap are somehow due to the negative pressure exerted by abiotic and biotic stresses on crops leading to low yields and abysmal product quality [151,170,171,172]. Pressure upon crops in the form of plant diseases, e.g., an increase in new pathogen strains in some areas of the world, has raised questions over current plant-protection measures and crop improvement strategies [173,174]. Over the past decades, plant biologists have targeted disease resistance to improve crop productivity. However, recent molecular findings have revealed diverse elements of plant immunity conferring the ability to identify and tackle specific pathogens [175,176]. Pathogens can overwhelm R genes through modification of their recognized effectors. Durable crop resistance can be achieved by employing crop R genes, exploiting effective receptors and target effectors. Cloned NB-LRR genes can facilitate their use in agriculture as molecular markers or by transgenic means. Several effector proteins identified by plant-immunity receptors can be screened against wild plant types to ascertain different resistance sources. A difference in NB-LRR expression levels can help in understanding disease resistance among wild and cultivated plants. Phosphorylation drives the start of PRR signaling, but PRR kinase activation mechanisms, along with particular phosphorylation actions mediating signal initiation, and interaction with downstream substrates, are clandestine. Like animals, deciphering the fate of activated PRRs and receptor kinases is very important in dissecting plant immunity. Functional evaluation and expression profiles of the diverse proteins constituting PRR complexes at the cell, or organ level are not completely known. One of the challenges for all researchers in this domain is revealing the development and activation of PRR complexes along with their succeeding links with downstream signaling networks for innate immunity. Additional studies involving the comprehensive integration of genomics with biochemical approaches can expand our understanding of PRR and NB-LRR-mediated regulatory mechanisms.

## Figures and Tables

**Table 1:** Many pattern-recognition receptors (PRRs) have been discovered in different plant species. PRRs can be categorized as receptor kinases or receptor-like proteins. Ligand recognition by PRRs leads to Pathogen Associated Molecular Pattern (PAMP)-Triggered Immunity (PTI). This table highlights PRR types along with their ligands/agonists.

Sr. No.	Family	Plant Source														PRR	Ligand/Agonist	Reference
		AT	BN	LJ	NB	OL	OS	SH	SL	SM	SP	SI	TA	VV	ZM			
**Receptor Kinase**																		
**1**	***LRRXII***															XA21	RaxX	[38]
**2**																FLS2	Flagellin (flg22 epitope)	[46]
**3**																FLS3	Flagellin (flgII-28 epitope)	[47]
**4**																EFR	EF-Tu (elf18 epitope)	[6]
**5**																XPS1	Xanthine/uracil permease (xup25 epitope)	[48]
**6**																CORE	csp22	[49]
**7**	***LRRXI***															PEPR1	Pep1-6	[50]
**8**																PEPR2	Pep1-2	[51]
**9**																RLK7	PIP1	[52]
**10**	***WAK***															WAK	Oligogalacturonides	[52]
**11**																Snn1/TaWAK	SnTox1	[53]
**12**	***LysM***															AtCERK1	Chitin	[54]
**13**																AtLYK5	Chitin	[55]
**14**																EPR3	Extracellular polysaccharides	[56]
**Receptor-Like Proteins**																		
**15**	***LRR***															Cf-2	Rcr3 protease (guarded to detect Avr2 and Gr-VAP1)	[57]
**16**																Cf-4	Avr4	[58]
**17**																Hcr9-4Eb	Avr4E	[59]
**18**																Cf-5	Avr5	[60]
**19**																Cf-9	HABS (guarded to detect Avr9)	[61]
**20**																Ve1	Ave1	[62]
**21**																LeEix2	EIX	[63]
**22**																LepR3/RLM2	AvrLm1and AvrLm2	[64]
**23**																RLP1/ReMAX	eMaxc	[65]
**24**																RLP23	nlp20	[66]
**25**																RLP30	SCFE1c	[67]
**26**																RLP42/RBPG1	EndoPG	[68]
**27**																RLP85/ELR	Elicitins	[69]
**28**																CSPR	csp22	[70]
**29**																CuRe1	Cuscuta factorc	[71]
**30**																I	Avr1/Six4	[55]
**31**	***LysM***															OsCEBiP	Chitin	[72]
**32**																OsLYP4 and OsLYP6	Peptidoglycans/chitin	[73]
**33**																AtLYM2	Chitin	[74]
**34**																AtLYM1 and AtLYM3	Peptidoglycans	[75]

**NOTE:**

**AT**

*Arabidopsis thaliana*

**BN**

*Brassica napus*

**LJ**

*Lotus japonicas*

**NB**

*Nicotiana benthamiana*

**OL**

*Oryza longistaminata*

**OS**

*Oryza sativa*

**SH**

*Solanum hirsutum*

**SL**

*Solanum lycopersicum*

**SM**

*Solanum microdontum*

**SP**

*Solanum pennellii*

**SI**

*Solanum pimpinellifolium*

**TA**

*Triticum aestivum*

**VV**

*Vitis vinifera*

***ZM***

*Zea mays*

**Table 2:** NB-LRRs from different plants and their role against diverse pathogens.

Gene	Plant	Function	Resistant Against	Reference
RFO1	*Arabidopsis thaliana*	Defense	*Fusarium*	[122]
RPW8	*Arabidopsis thaliana*	Defense	*Powdery mildew*	[123]
WRR4	*Arabidopsis thaliana*	Defense	*Albugo*	[124]
RCT1	*Medicago truncatula*	Defense	*Anthracnose*	[125]
NBS191	*Arachis duranensis*	Defense	*A. flavus*	[126]
QRR1	*Medicago truncatula*	Defense	*Ralstonia solanacearum*	[127]
Rpsar-1	*Phaseolus vulgaris*	Defense	*P. syringae*	[128].

**Table 3:** Many NB-LRRs were predicted with and without NLS. Irrespective of presence of a definite NLS, some NB-LRRs have their subcellular localization in nucleus and different sites.

Organism	NB-LRR Type	NLS		Localization Site				Reference
		YES	NO	Nucleus	Cytoplasm	Plasma Membrane	Others	
*Arabidopsis thaliana*	RRS1-R	√						[146]
	RPS4	√						[137]
	RPM1	√						[139]
	RPS5		√					[147]
*Linum usitatissimum*	L6		√					[148]
	M		√					[148]
*Hordeum vulgare*	MLa10		√					[149]
	MLa1		√					[149]
*Nicotiana glutinosa*	N	√						[150]
